# Proteogenomics Reveals Enriched Ribosome Assembly and Protein Translation in *Pectoralis major* of High Feed Efficiency Pedigree Broiler Males

**DOI:** 10.3389/fphys.2017.00306

**Published:** 2017-05-16

**Authors:** Walter G. Bottje, Kentu Lassiter, Alissa Piekarski-Welsher, Sami Dridi, Antonio Reverter, Nicholas J. Hudson, Byung-Whi Kong

**Affiliations:** ^1^Department of Poultry Science, Center of Excellence for Poultry Science, University of ArkansasFayetteville, AR, USA; ^2^Computational and Systems Biology, Agriculture and Food (CSIRO)St. Lucia, QLD, Australia; ^3^Animal Science, School of Agriculture and Food Science, University of QueenslandGatton, QLD, Australia

**Keywords:** feed efficiency, pedigree broiler male, breast muscle, proteomics, transcriptomics, ribosome assembly

## Abstract

**Background:** In production animal agriculture, the cost of feed represents 60–70% of the total cost of raising an animal to market weight. Thus, development of viable biomarkers for feed efficiency (FE, g gain/g feed) to assist in genetic selection of breeding stock remains an important goal in commercial breeding programs.

**Methods:** Global gene (cDNA microarray, RNAseq) and protein expression (shotgun proteomics) analyses have been conducted on breast muscle samples obtained from pedigree broiler males (PedM) exhibiting high and low FE phenotypes. Using the entire datasets (i.e., no cutoffs for significance or fold difference in expression) the number of genes or proteins that were expressed numerically higher or lower in the high FE compared to the low FE phenotype for key terms or functions, e.g., ribosomal, mitochondrial ribosomal, tRNA, RNA binding motif, RNA polymerase, small nuclear ribonucleoprotein, and protein tyrosine phosphatase, were determined. Bionomial distribution analysis (exact) was then conducted on these datasets to determine significance between numerically up or down expression.

**Results:** Processes associated with mitochondrial proteome expression (e.g., mitochondrial ribosomal proteins, mitochondrial transcription, mitochondrial tRNA, and translation) were enriched in breast muscle from the high FE compared to the low FE pedigree male broiler phenotype. Furthermore, the high FE phenotype exhibited enrichment of ribosome assembly (e.g., RNA polymerase, mitochondrial and cytosolic ribosomes, small, and heterogeneous nuclear ribonucleoproteins), as well as nuclear transport and protein translation processes compared to the low FE phenotype. Quality control processes (proteosomes and autophagy) were also enriched in the high FE phenotype. In contrast, the low FE phenotype exhibited enrichment of cytoskeletal proteins, protein tyrosine phosphatases, and tyrosine kinases compared to the high FE phenotype. These results suggest that processes of mitochondrial and cytosolic ribosomal construction, activity, and protein translation would be enhanced in high FE breast muscle, and that phosphorylation of tyrosine moieties of proteins could be prolonged in the high compared to low FE phenotype. The results indicate the presence of a proteogenomic architecture that could enhance ribosome construction, protein translation, and quality control processes and contribute to the phenotypic expression of feed efficiency in this PedM broiler model.

## Introduction

Continued improvement in animal agriculture production efficiency is critical for maintaining sustainable poultry and livestock production. Simultaneously, enhancing feed efficiency (FE) helps meet the protein needs of an ever increasing human population, while making better use of available resources. As animal feed comprises 60–70% of total cost of raising an animal to market weight across poultry and livestock (see Arthur and Herd, 2005), FE remains an important genetic trait for commercial breeding in poultry and livestock. At the cellular level, the efficiency of how energy is produced and utilized ultimately contributes to cellular utilization efficiency.

In most animal species, skeletal muscle accounts for roughly 50% of body mass. When considered as a single organ, skeletal muscle contributes between 25 and 40% of overall basal metabolic rate (Brand, 1990; Zurlo et al., 1990; Rolfe et al., 1999), 25% of which is attributed to one component of mitochondrial function; i.e., proton leak (Rolfe and Brand, 1996, 1997). A clear link between breast muscle mitochondrial physiology and FE has been reported (Bottje et al., 2002; Bottje and Carstens, 2009). Thus, skeletal muscle mitochondria can play a substantive role in overall efficiency in animals.

In global gene and protein expression studies, emphasis is placed on using cutoff values for fold differences (e.g., >1.3-fold, *P* < 0.05) between groups which produces well defined, differentially expressed genes or proteins datasets that are verifiable, manageable, and can be discussed in a cohesive manner. This approach enhances understanding of fundamental mechanisms associated with given condition, but a tremendous amount of potentially informative data is discarded from further consideration. This is particularly true for high throughput genomic data where the number of assays (e.g., cDNA probes or RNA reads) vastly exceeds the number of biological replicates. In these cases multiple testing penalties can be very severe and may lead to few, if any, significant differences being identified. Here we present an alternative approach that may especially lend itself to samples with subtle phenotypes such as feed efficiency within a single genetic line. We argue that individually, small effects can be considered significant if they combine cumulatively in a given biological process or pathway.

To obtain more information from global expression datasets, phenotypic and regulatory impact (PIF and RIF) analyses were developed to identify molecules that have a large effect on a given phenotype due to their interconnectedness with many cellular processes, but may not be differentially expressed (e.g., Hudson et al., 2009, 2012). Using RIF analysis, we reported that progesterone signaling plays a role in the phenotypic expression of FE in the skeletal muscle of pedigree male (PedM) broilers (Bottje et al., 2017). It was also reported that the mitochondrial transcriptome and proteome were elevated in the high FE phenotype (based on binomial analysis of numbers of transcripts or proteins that were numerically or significantly higher in high vs. low FE PedM broiler phenotype, Kong B. et al., 2016; Bottje et al., 2017). These studies represent a precedent for using binomial statistics at a particular process level (all mRNA encoding the mitochondrial proteome and all proteins present in the mitochondria) to gather insights that would not have been possible with traditional methods. The common finding by both independent approaches (mRNA and protein) supports the utility of using binomial statistics in this manner.

It is not clear whether the elevation of the mitoproteome we detected in the high FE PedM broilers is a consequence of an elevated mitochondrial content, an elevated mitochondrial activity or some weighted combination of the two. For example, Kong R. S. et al. (2016) recently discovered that more efficient cattle exhibited elevated mitochondrial gene expression but a lower mitochondrial content in rumen epithelia. In order for the mitoproteome to be elevated in the high FE phenotype (Kong B. et al., 2016), we hypothesize that, not only must mitoproteome gene expression be elevated (Bottje et al., 2017), there must also be supportive architecture involving, cytosolic and mitochondrial ribosome expression, RNA synthesis, and specialized ribonucleoproteins such as that provided in a review by Wahl et al. (2009) that would foster mitoproteome expression. Thus, the major goal of this data mining study was to assess the numbers of genes and proteins in global expression datasets in muscle obtained from high and low FE PedM broiler phenotypes associated with upstream components requisite for protein synthesis.

## Materials and methods

### Ethics statement

The present study was conducted in accordance with the recommendations in the guide for the care and use of laboratory animals of the National Institutes of Health. All procedures for animal care were reviewed and approved by the University of Arkansas Institutional Animal Care and Use Committee (IACUC): Protocol #14012.

### Tissues-animals

The global gene and protein expression data examined in this study were obtained previously (Kong et al., 2011; Kong B. et al., 2016; Bottje et al., 2017). The data were all obtained from the same set of breast muscle samples from pedigree male (PedM) broilers individually phenotyped for feed efficiency (FE) using procedures previously described (Bottje et al., 2002). Briefly, FE (amount of body weight gain/amount of feed consumed) was determined between 6 and 7 week of age on a group of 100 PedM broilers housed in individual cages in the same environment. All birds received the same corn-soybean based diet (20.5% protein, 3,280 kcal/kg) and water *ad libitum* during the 1 week phenotyping period (from 6 to 7 week of age). From this group of 100 birds, those exhibiting the highest and lowest FE (*n* = 6 per group) were obtained. The average weight gain (g), feed intake (FI, g), and FE (gain/feed) for the high and low FE groups is provided in Table 1. The same tissue samples were analyzed in both the microarray (Kong et al., 2011) and RNAseq (Bottje et al., 2017) studies whereas a subset of these samples (*n* = 4/group) were analyzed in the proteomic study (Kong B. et al., 2016). In Table 1, it is apparent that the high FE PedM broilers achieved better FE by gaining more weight on the same amount of feed consumption compared to the low FE phenotype. After humane killing, breast muscle samples were obtained, flash frozen in liquid nitrogen and stored at −80°C until analysis.

**Table 1:** **Body weight gain (g), feed intake (FI, g), and feed efficiency (FE, gain/FI) of pedigree broiler males (PedM) exhibiting high FE and low FE phenotypes^**a**^**.

	***n***	**Body wt. gain (g)**	**Feed intake (g)**	**Feed efficiency (g gain/g feed intake)**
**MICROARRAY (Kong et al., 2011) and RNAseq (Bottje et al., 2017)^b^**				
PedM High FE	6	641 ± 20^*^	973 ± 33	0.659 ± 0.011^*^
PedM Low FE	6	485 ± 17	1048 ± 34	0.462 ± 0.006
**PROTEOMICS (Kong B. et al., 2016)^b^**				
PedM High FE	4	660 ± 18^*^	1019 ± 21	0.648 ± 0.007^*^
PedM Low FE	4	491 ± 26	1075 ± 45	0.456 ± 0.006

a *Values represent the mean + SE*.

b *The same muscle tissues (n = 6/group) were analyzed in the microarray and RNAseq studies and a subset of the same muscle tissues (n = 4/group) were analyzed in the proteomics*.

* *Mean value is greater (P < 0.05)*.

### Global expression analysis

Detailed descriptions of global gene expression analysis by cDNA microarray and RNAseq are provided in Kong et al. (2011) and Bottje et al. (2017), respectively. Procedures for global protein expression analysis (shotgun proteomics) are provided in Kong B. et al. (2016). In the cDNA microarray study (Kong et al., 2011), following RNA extraction equal amounts of RNA obtained from 6 separate muscle samples for the high and low FE groups were pooled and hybridized on a 4 × 44 K Agilent chicken oligo microarray (array ID: 015068; Agilent Technologies, Inc.) with four replicates of each group in the microarray analysis. An argument for the use of pooled samples in the microarray study was provided in a companion paper (Bottje et al., 2012):

“*In a comparison of pooled vs. individual sample microarray analysis, Jolly et al. (2005) concluded that ‘while the two approaches to running microarray chips were comparable…the individual analysis revealed subtle changes that affect interpretation of the experiment that were lost in the pooled analysis and important for mechanistic understanding’. However, justification for use of pooled samples was provided by Kendziorski et al. (2005) who indicated that pooling samples for microarray analysis also minimizes subject-to-subject variation that “is often desirable when primary interest is not on the individual but rather on characteristics of the population from which certain individuals are obtained (e.g., identifying biomarkers or expression patterns common across individuals).” Similarly, in the context of the present paper, we are more interested in looking for gene expression patterns across individuals in the high and low FE phenotypes according to Kendziorski et al. (2005) at the potential expense of losing some mechanistic understanding as indicated by Jolly et al. (2005)*.”

For the RNAseq analysis, RNA was isolated from 6 breast muscle samples per group and analyzed with an Illumina HiSeq using 2 × 100 bp paired end read sequencing at the Research Support Facility at Michigan State University (East Lansing, MI) on an Illumina HiSeq using 100 base paired end read sequencing. Shotgun proteomics analysis was conducted on breast muscle from a total of 4 high and 4 low FE samples by in-gel trypsin digestion and tandem mass spectrometry (MS/MS) at the University of Arkansas Medical Science (UAMS) Proteomics Core Lab (Little Rock, AR).

### Statistical analysis

The number of genes or proteins associated with a specific process were determined by compiling a list of molecules after searching each dataset for specific terms that are provided in Tables 2–**5**. The numbers of molecules in which mean values were numerically higher (H) or lower (L) in breast muscle of the high FE compared to the low FE PedM phenotype were determined and used in the exact binomial distribution analysis test offered in the 2010 version of Microsoft Excel™. In this study there was no gating of terms based on significant or fold difference in expression for a given transcript or protein. The mean values for the proteomics dataset (Kong B. et al., 2016) were based on 4 samples per FE phenotype and were a subset obtained from the same 6 samples that were analyzed per FE phenotype in the microarray (Kong et al., 2011) and RNAseq (Bottje et al., 2017) studies.

**Table 2:** **Mitochondrial ribosomal protein gene expression list obtained from the RNAseq dataset of breast muscle tissue generated by Bottje et al. (**2017**) showing log_**2**_ high feed efficiency—log_**2**_ low feed efficiency (M), the gene symbol and the gene name**.

**M**	**Symbol**	**Entrez Gene Name**
1.13	MRPL55	Mitochondrial ribosomal protein L55
0.72	MRPL24	Mitochondrial ribosomal protein L24
0.71	MRPS18C	Mitochondrial ribosomal protein S18C
0.53	MRPL9	Mitochondrial ribosomal protein L9
0.52	MRPL17	Mitochondrial ribosomal protein L17
0.44	MRPL18	Mitochondrial ribosomal protein L18
0.36	MRPL42	Mitochondrial ribosomal protein L42
0.36	MRPS11	Mitochondrial ribosomal protein S11
0.36	MRPS22	Mitochondrial ribosomal protein S22
0.36	MRPS35	Mitochondrial ribosomal protein S35
0.35	MRPL21	Mitochondrial ribosomal protein L21
0.34	MRPL44	Mitochondrial ribosomal protein L44
0.33	MRPL2	Mitochondrial ribosomal protein L2
0.33	MRPL47	Mitochondrial ribosomal protein L47
0.32	MRPS5	Mitochondrial ribosomal protein S5
0.30	MRPS21	Mitochondrial ribosomal protein S21
0.30	MRPS23	Mitochondrial ribosomal protein S23
0.29	MRPL16	Mitochondrial ribosomal protein L16
0.29	MRPL35	Mitochondrial ribosomal protein L35
0.29	MRPS31	Mitochondrial ribosomal protein S31
0.28	MRPL3	Mitochondrial ribosomal protein L3
0.27	MRPL38	Mitochondrial ribosomal protein L38
0.26	MRPL32	Mitochondrial ribosomal protein L32
0.26	MRPL54	Mitochondrial ribosomal protein L54
0.26	MRPS26	Mitochondrial ribosomal protein S26
0.25	MRPL46	Mitochondrial ribosomal protein L46
0.24	MRPL51	Mitochondrial ribosomal protein L51
0.24	MRPS25	Mitochondrial ribosomal protein S25
0.23	MRPS2	Mitochondrial ribosomal protein S2
0.22	MRPL50	Mitochondrial ribosomal protein L50
0.20	MRPL10	Mitochondrial ribosomal protein L10
0.20	MRPL23	Mitochondrial ribosomal protein L23
0.17	MRPL39	Mitochondrial ribosomal protein L39
0.17	MRPL48	Mitochondrial ribosomal protein L48
0.17	MRPS18A	Mitochondrial ribosomal protein S18A
0.16	MRPL40	Mitochondrial ribosomal protein L40
0.16	MRPS36	Mitochondrial ribosomal protein S36
0.15	MRPS9	Mitochondrial ribosomal protein S9
0.14	MRPL14	Mitochondrial ribosomal protein L14
0.13	MRPS6	Mitochondrial ribosomal protein S6
0.12	MRPS34	Mitochondrial ribosomal protein S34
0.11	MRPL1	Mitochondrial ribosomal protein L1
0.11	MRPL20	Mitochondrial ribosomal protein L20
0.11	MRPS15	Mitochondrial ribosomal protein S15
0.10	MRPL28	Mitochondrial ribosomal protein L28
0.09	MRPL53	Mitochondrial ribosomal protein L53
0.08	MRPL37	Mitochondrial ribosomal protein L37
0.06	MRPL22	Mitochondrial ribosomal protein L22
0.06	MRPS7	Mitochondrial ribosomal protein S7
0.05	MRPS10	Mitochondrial ribosomal protein S10
0.04	MRPS14	Mitochondrial ribosomal protein S14
0.04	MRPS30	Mitochondrial ribosomal protein S30
0.02	MRPL15	Mitochondrial ribosomal protein L15
0.00	MRPS27	Mitochondrial ribosomal protein S27
−0.01	MRPL41	Mitochondrial ribosomal protein L41
−0.01	MRPS33	Mitochondrial ribosomal protein S33
−0.03	MRPL13	Mitochondrial ribosomal protein L13
−0.07	MRPS17	Mitochondrial ribosomal protein S17

## Results and discussion

### Mitochondrial ribosome proteins are enriched in high FE breast muscle

We reported that mitoproteome expression was enriched in breast muscle of high FE compared to low FE PedM broilers (Kong B. et al., 2016; Bottje et al., 2017). For this to occur, we hypothesize that a genomic architecture must exist that would foster mitochondrial protein synthesis in the high FE phenotype including production of mitochondrial ribosomes (e.g., Graack et al., 1999) and components of RNA synthesis, ribosome assembly, and protein translating activities (e.g., Wahl et al., 2009). A list of all genes associated with the term “mitochondrial ribosomal protein” in the RNAseq dataset (Bottje et al., 2017), is provided in Table 2 as an example of data used in binomial distribution analysis in Tables 3–**5**. Instances in which gene and protein expression differences between high and low FE groups were 0 were rare; e.g., only one gene, MRPS27, out of 59 total mitochondrial ribosomal protein transcripts listed in Table 1. Percentages of genes or proteins that exhibited no difference in mean expression between high and low FE groups in global expression datasets were 0.23%, (4 of 1,806 proteins; Kong B. et al., 2016) and 1.52% (167 of 10,981 transcripts; Bottje et al., 2017).

**Table 3:** **Numbers of genes and proteins associated with mitochondrial protein synthesis (ribosomes, transcription, and tRNA) as well as transport of molecules into the mitochondria that were higher (H) or lower (L) in breast muscle of high feed efficiency (FE) compared to low FE pedigree broiler male for the terms provided obtained from global expression microarray, proteomics, and RNAseq datasets and for combined in all three global expression datasets**.

**Terms**	**Microarray^a^ —**			**Proteomics^b^ —**			**RNAseq^c^ —**		
	**H**	**L**	P^*^	**H**	**L**	P^*^	**H**	**L**	P^*^
Mitochondrial ribosomal proteins	18	6	0.008	0	1		55	4	8 × 10^−13^
TOMM^d^	0	0		2	1		2	2	
TIMM^e^	1	1		0	0		9	0	0.0029
Mitochondrial transcription	2	0		0	0		5	1	0.0938
Mitochondrial tRNA	1	0		1	0		14	2	0.0018
Mitochondrial ribosome, translational, processing, peptidase	4	0	0.0625	0	0		6	2	0.1093

a *cDNA microarray —Kong et al. (2011)*.

b *Shotgun proteomics—Kong B. et al. (2016)*.

c *RNAseq–Bottje et al. (2017)*.

* *Binomial (exact) P values for H vs. L in each dataset*.

d *Transport protein outer mitochondrial membrane*.

e *Transport protein inner mitochondrial membrane*.

**Figure 1:**
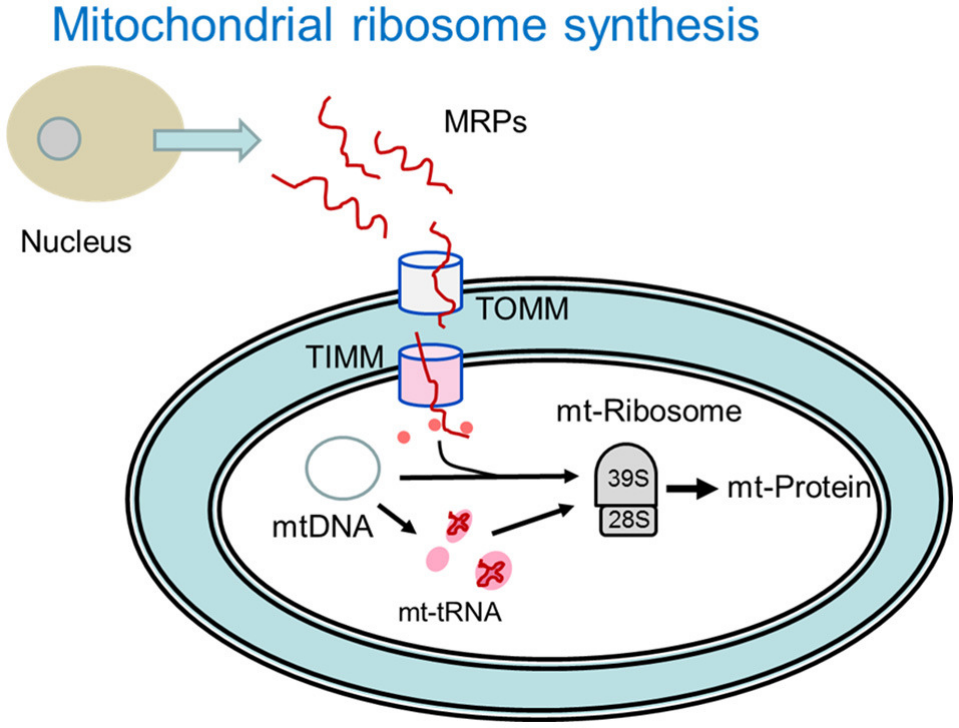
**Depiction of mitochondrial ribosomal protein (MRP) import and synthesis of mitochondrial (mt) proteins encoded by mitochondrial DNA (mtDNA) enriched in the high feed efficiency pedigree male broiler phenotype**. MRPs are synthesized in the nucleus and imported into the mitochondria through outer mitochondrial membrane transport (TOMM) proteins and inner mitochondrial mitochondrial membrane proteins (TIMM). Once inside the mitochondria, mt transfer RNAs (mt-tRNA) encoded by mtDNA facilitate protein translation that takes place on the mitochondrial 39S and 28S ribosomal subunits. Pink or red color denotes processes that were enriched in the high feed efficiency pedigree broiler male as indicated in Table 3 (See text for more details).

Binomial analysis associated with mitochondrial protein expression machinery is provided in Table 3 and depicted in Figure 1. Enrichments favoring the high FE phenotype were observed for genes encoding mitochondrial ribosomal proteins, mitochondrial tRNA, mitochondrial transcription and for inner mitochondrial membrane transport proteins. Mitochondrial ribosomes (55*S*) are very different from cytoplasmic (80*S*) and bacterial ribosomes (70S) (Avadhani and Buetow, 1974; O'Brien, 2002). Mitochondrial ribosomes have a greater protein content compared to bacterial and cytoplasmic ribosomes (Sylvester et al., 2004) as well as proteins that are unique to the mitochondrial ribosomes (Graack et al., 1999). In mammals, all mitochondrial ribosomal proteins (~80) are encoded by nuclear genes and translated on cytoplasmic ribosomes prior to import into the mitochondria through transport proteins on the outer (TOMM) and inner (TIMM) mitochondrial membranes (Scheiber and O'Brien, 1985; O'Brien, 2002; Fox, 2012). While there was no difference between FE groups for TOMM, 10 of 11 TIMM transcripts that were detected in the data were higher in the high FE phenotype (Table 2). Once inside, mitochondrial ribosomal proteins assemble into the 39S and 28S ribosomes that translate messages from mitochondrial (mt) DNA encoded genes that include 13 electron transport chain proteins, the 16S and 12S rRNA genes and 22 tRNA genes that are essential for synthesis of mitochondrial proteins (Wallace, 1999; Sue and Schon, 2000). Enrichment of mitochondrial transcription, mitochondrial tRNA and for mitochondrial translation and processing of mtDNA encoded proteins was evident in the high FE phenotype (Table 3) and consistent with the enriched mitoproteome in the high FE phenotype (Kong B. et al., 2016; Bottje et al., 2017).

The data presented in Table 3 highlights a well-established short coming of proteomic vs. transcriptomic analyses. Extraction of mRNA is a largely unbiased procedure that occurs with equal efficiency irrespective of the function of the encoded proteins. For all intents and purposes, every mRNA in a given tissue sample will be detected by modern screening approaches. On the other hand, proteomic extractions are very sensitive to the biochemical properties of the individual proteins, such as solubility, which can differ markedly both within and between organelles in general, and especially with the mitoproteome that contains a large number of proteins with hydrophobic regions, such as those associated with the electron transport chain (Lescuyer et al., 2003). In addition, proteomic analyses tend to be biased toward abundant structural proteins and may fail to identify less abundant but very important regulatory proteins such as transcription factors. In our case, the RNAseq and microarray analyses detected 103 and 30 transcripts encoding mitochondrial proteins, respectively, compared to 5 mitochondrial proteins for the terms shown in Table 3. This discrepancy is also related to the total numbers of molecules that were detected; ~1,800 proteins compared to more than 4,000 and 10,000 gene transcripts in the microarray and RNAseq datasets, respectively.

### Ribosome assembly machinery, nuclear transport, and protein translation enrichment in high FE muscle

Over 1,500 mitochondrial proteins are encoded by nuclear (*n*) DNA and imported into the mitochondria, to form enzyme complexes of the electron transport chain, Kreb cycle, and other mitochondrial processes (Wallace, 1999; Sue and Schon, 2000; Lescuyer et al., 2003; Taylor et al., 2003; Chevallet et al., 2006). Thus, mitoproteome expression is highly dependent on nuclear transcription and protein translation taking place on cytoplasmic ribosomes. Based on a review by Wahl et al. (2009), the global expression datasets were searched for terms associated with RNA synthesis, ribosome assembly, protein translating activity, small nuclear (sn) ribonucleoproteins (RNPs) and heterogenous (hn) SNPs, and expression of nuclear pore molecules including nucleoporins, karyopherins, importins, and exportins (Table 4). Transcription of ribosomal (r), messenger (m), and transfer (t) RNAs is initiated by RNA polymerases I, II, and III (Roeder and Rutter, 1969, 1970; Sentenac, 1985). Although, there was no difference in the microarray and proteomic data, RNA polymerase was enriched in the high FE phenotype in the RNAseq data (Table 4). Binomial analysis of the combined data, however, revealed significant skew of RNA polymerase toward the high FE phenotype (data not shown). The findings suggest that RNA polymerase was enriched in the high FE phenotype but further study is needed to determine if this difference would translate to higher RNA polymerase activity in the high FE phenotype.

**Table 4:** **Numbers of genes and proteins associated with transcription, pre-mRNA processing, and ribosomal assembly that were higher (H) or lower (L) in breast muscle of high feed efficiency (FE) compared to low FE pedigree broiler male for the terms provided obtained from global expression microarray, proteomics, and RNAseq datasets and for numbers of all three global expression datasets combined**.

	**Microarray**^**a**^ **—**			**Proteomics**^**b**^ **—**			**RNAseq**^**c**^ **—**		
**Terms**	**H**	**L**	P^*^	**H**	**L**	P^*^	**H**	**L**	P^*^
RNA Polymerase	14	13		1	2		28	8	0.0004
Transcription	16	22	0.0812	3	1		94	69	0.0092
TATA Box, T-box	6	4		0	0		19	8	0.0165
Small nuclear (sn) ribonucleoproteins (RNP) and heterogeneous (hn) RNP	10	1	0.0054	4	1		12	1	0.0016
Spliceosome, helicase,	6	1	0.0547	4	0	0.0625	27	11	0.0044
pre mRNA processing									
RNA Binding Motif	11	8		0	0		39	13	0.0001
Ribosome-Ribosomal	8	1	0.0017	41	4	4 × 10^−9^	57	49	0.0573
Transfer RNA (tRNA)	8	8		6	0	0.0156	64	11	1.0 × 10^−10^
Translation (Eukaryotic initiation-elongation)	12	11		12	7	0.0961	43	13	3.0 × 10^−5^
Karyopherins (Nuclear transport proteins)	5	6	0.0537	1	0		24	15	0.0457
(Nucleoporins, Karyopherins, Importins, Exportins)									
Small ubiquitin-like modifier (SUMO)	1	1		1	1		9	2	0.0269
(peptidase, isopeptidase, ligase)									

a *cDNA microarray—Kong et al. (2011)*.

b *Shotgun proteomics—Kong B. et al. (2016)*.

c *RNAseq—Bottje et al. (2017)*.

* *Binomial (exact) P values for H vs. L (higher vs. lower) in each dataset*.

*P values not underlined indicate a significant bionomial skew in high FE numbers*.

*P values that are underlined indicate a significant skew in low FE numbers*.

*P values that are blank are indicative of values > 0.10*.

When “transcription” was used as the search term, binomial distribution analysis revealed a marginal enrichment in the low FE phenotype enrichment (*P* = 0.08) in the microarray data but a significant enrichment in the high FE phenotype with the RNAseq dataset (Table 4). The reason for the discrepancy in the two transcript analyses is not apparent at this time. Typically, transcription promotors contain a sequence that recognizes the promotor nucleotide base sequences (TATA), and recruits TATA-binding proteins (TBP) to the site on DNA where transcription begins following RNA polymerase activity (Cormack and Struhl, 1992; Schultz et al., 1992; Pugh, 2000; Davidson, 2003). The TATA box (and T-box) is considered to be the core promoter sequence and the binding site of general transcription factors and histones; the binding of a transcription factor blocks the binding of a histone and vice versa. Higher numbers of genes associated with TATA box and T-box may serve to facilitate gene transcription in the high FE phenotype (Table 4).

The removal of introns in the nucleus is accomplished by coordinated activities involving proteins and RNAs found in the spliceosome (see Wahl et al., 2009). In this pre-mRNA processing step, and during transcription of mRNA and tRNA prior to export from the nucleolus and nucleus, are numerous RNA binding proteins targeting RNA binding motifs. Spliceosome, helicase, and pre-mRNA processing components were all enriched in the high FE phenotype (Table 4).

As protein synthesis strongly correlates with cellular rRNA and tRNA content, cells need to sustain high rates of RNA polymerase I and III transcription to synthesize as many as 2 million ribosomes per cell (Drygin et al., 2010). Ribosomes and tRNA enrichment was observed in the high FE phenotype (Table 4). Although, no mitochondrial ribosomal proteins were detected with shotgun proteomics (Table 3), many ribosome and ribosomal-related proteins were detected and were enriched in the high FE phenotype (Table 4). Protein translation is facilitated by eukaryotic initiation and elongation proteins. While there was no difference in the numbers of initiation and elongation protein transcripts in the microarray dataset, marginal and significant enrichments were observed in the proteomics and RNAseq datasets, respectively (Table 4). Collectively, these results indicate that requisite architecture for transcription and translation processes were enriched in breast muscle of the high FE compared to the low FE PedM broiler.

The nucleopore complex is vital in separating the nucleoplasm from the cytoplasm, and in trafficking of RNA components (e.g., mRNAs, tRNAs, and rRNAs) out of the nucleus, and cytoplasmic synthesized proteins into the nucleus that are requisite for RNA synthesis outlined above. The nucleopore complex is comprised of a large family of karyopherin proteins with several protein subfamilies; e.g., nucleoporins, importins, and exportins (see review by Wente and Rout, 2010). The action of these proteins in nuclear transport are modulated by small ubiquitin-like modifying (SUMO) proteins that are in turn regulated by SUMO peptidase and ligase activities. The high FE phenotype exhibited a significant enrichment of nuclear transport proteins as well as with SUMO proteins for the RNAseq data analysis (Table 4) that could be hypothesized to favor nuclear transport and/or regulation of RNA and protein transport between the nuclear and cytoplasm compartments.

### Enrichment of cytoarchitecture-muscle fibers in low FE muscle

The low numbers of proteins relative to the number of transcripts observed in the proteomic dataset in Tables 2–4 could be due to fewer proteins being detected compared to transcriptomic analyses, as well as the technical limitations of protein detection as mentioned previously. In the microarray data, we reported a general down-regulation of many transcripts associated with cytoskeletal architecture and/or muscle fibers (Kong et al., 2011; Bottje and Kong, 2013) that was also observed in the RNAseq study (Bottje et al., 2017). The implications of these findings on muscle cytoarchitecture in the high and low FE groups were discussed in detail in Bottje et al. (2017) and won't be repeated here. A search for actin (ACTA), myosin (MYH) and troponin (TNNC) yielded results indicating enrichment of these molecules in the low FE muscle phenotype across all three global expression datasets (Table 5). The numbers of actin, myosin and troponin in the proteomics dataset were similar to numbers of transcripts in the microarray dataset and may reflect that abundances of these proteins were much greater (e.g., >100 spectral counts) compared to normalized spectral counts ranging from 5 to 30 for proteins listed in Tables 3, 4.

**Table 5:** **Numbers of genes and proteins associated with cytoskeletal-muscle fibers, phosphorylation regulation, cellular quality control mechanisms (proteosome and autophagy) that were higher (H) or lower (L) in breast muscle of high feed efficiency (FE) compared to low FE pedigree broiler male for the terms provided obtained from global expression microarray, proteomics, and RNAseq datasets and for numbers of all three global expression datasets combined**.

	**Microarray**^**a**^			**Proteomics**^**b**^			**RNAseq**^**c**^			
**Term**	**H**	**L**	P^*^	**H**	**L**	P^*^	**H**	**L**	P^*^	**H**
**PHOSPHORYLATION**										
Cytoskeletal-muscle fibers^d^	9	20	0.0004	7	21	0.0044	21	42	0.0030	35
Protein tyrosine phosphatase	2	1		2	0		5	27	0.0020	9
Protein phosphatase	4	2		7	1	0.0313	30	24	0.0778	41
Tyrosine kinase	6	4		1	0		11	31	0.001	17
**QUALITY CONTROL**										
Proteosome	7	3	0.1172	21	4	0.0004	31	4	2E-06	59
Autophagy	6	2	0.1093	4	0	0.0625	36	15	0.0014	49

a *cDNA microarray—Kong et al. (2011)*.

b *Shotgun proteomics—Kong B. et al. (2016)*.

c *RNAseq—Bottje et al. (2017)*.

d *Actin (ACTA), myosin (MYH), troponin (TNNT)*.

* *Binomial (exact) P values for H vs. L in each dataset. Blanks are indicative of P > 0.10*.

*P values not underlined indicate a significant bionomial skew in high FE*.

*P values that are underlined indicate a significant skew in low FE*.

*P values that are blank are indicative of values > 0.10*.

### Phosphorylation regulation

Activation or deactivation of enzymes and numerous cascade pathways are carried out by a large number of diverse phosphatases and kinases (e.g., see reviews: Hunter and Cooper, 1985; Hubbard and Till, 2000; Vishup, 2000; Mustelin, 2007). Although, we did not do an exhaustive search in these areas, a few are provided indicating that certain components of cell metabolism were enhanced in the low FE phenotype. Whereas protein tyrosine phosphatase and protein tyrosine kinase families were enriched in the low FE phenotype, protein phosphatase was enriched in the high FE phenotype (Table 5). Because of the diverse nature of phosphorylation reactions, it is very difficult to speculate the role that this apparent differential enrichment of phosphatases and kinases have in contributing to a high or low FE phenotype. Nonetheless, it is apparent that there are distinct differences and additional research is needed in order to fully characterize the role that individual enzymes might have in the phenotypic expression of feed efficiency.

### Enrichment of quality control processes: proteosome and autophagy

Interestingly, enrichment of protein and/or organelle degradation associated with cellular quality control mechanisms of proteosome and autophagy processes was observed in the high FE phenotype (Table 5). At the outset, these processes appear to be a counterproductive to cellular efficiency as protein degradation and resynthesis requires tremendous amounts of energy. Based on significant differences in gene expression, we had previously hypothesized that proteosomal activity was increased in the high FE phenotype (Bottje et al., 2014). This hypothesis is supported in the present study in which both proteomic and transcriptomic data indicate proteosomal enrichment in the high FE phenotype (Table 5). Possibly, enhanced proteosomal activity in the high FE muscle serves to recycle proteins at a more rapid rate, keeping the overall functionality of proteins optimal in high FE. Furthermore, the proteosome is not only involved in protein hydrolysis and protein resynthesis, it may also support a high degree of protein functionality through refolding when the tertiary structure becomes misaligned (Hershko and Ciechanover, 1986; Ciechanover, 1998; Lecker et al., 2006). An additional role of proteosomal activity has also been proposed as an important component of transcription (see review, Muratani and Tansey, 2003). In this model, the ubiquitin-proteosome complex interacts with general transcription machinery that includes ubiquitinylation of RNA polymerase II that in turn recruits the 26S proteosome (composed of the 19S and 20S proteosome). The 26S proteosome then functions to maintain optimal promoter-activator interactions between the RNA polymerase II-26S proteosome complex. Based on gene expression in the microarray dataset, we had proposed that formation of the RNA polymerase II pre-initiation complex would favor enhanced transcription processes in the high FE phenotype (Bottje et al., 2014). *In vitro*, the proteosome has been shown to bind to unfolded proteins, stimulate refolding, and release the protein back into the cytosol in its original tertiary/optimal structure form (Braun et al., 1999; Strickland et al., 2000; Glickman and Cienhanover, 2002). The proteosome has also been shown to inhibit the aggregation of misfolded proteins in the cytosol (Braun et al., 1999). Thus, the enrichment of proteosomes in the high FE could help maintain optimal functionality of proteins in the cell either by rapid degradation and resynthesis of damaged proteins or maintaining optimal protein tertiary structure. Interestingly, a new homeostatic role of mitochondria in the cell may be in complementing proteosome activity through degradation of protein aggregates formed in the cytosol that are imported into the mitochondria (Ruan et al., 2017). Thus, it is possible that the enhanced expression of the mitoproteome (Kong B. et al., 2016; Bottje et al., 2017) could also function in quality control of damaged components in the cell.

Autophagy (self-eating) is another degradative repair process that degrades damaged structures ranging from proteins to entire organelles (e.g., mitochondria, endoplasmic reticulum; Klionsky, 2005; Massey et al., 2006; Tasdemir et al., 2007; Levine and Kroemer, 2008). During starvation, autophagy can generate energy via protein and lipid catabolism and remove damaged components followed by recycling of amino acids for new *de novo* synthesis of proteins (e.g., Hamasaki et al., 2008). Although, this system has been well characterized in organisms ranging from yeast to mammals, the first characterization of autophagy expression in avian tissues and cells was only reported recently (Piekarski et al., 2014). Evidence that components of autophagy pathway were up-regulated in the high FE phenotype (from the same tissues that were used in the global gene and protein expression datasets in the current study) has been reported (Piekarski, 2015).

### Summary

**Figure 2:**
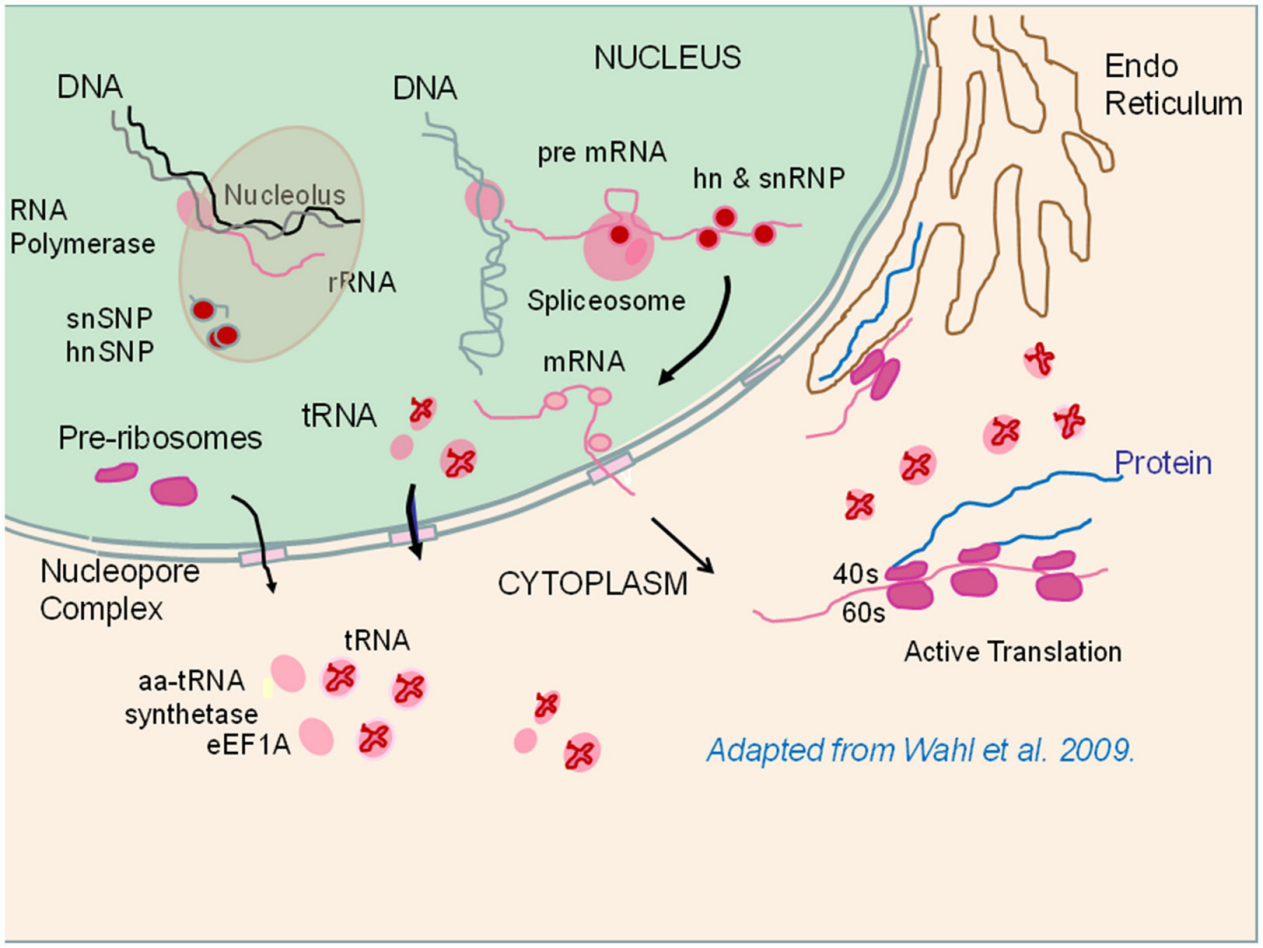
**Depiction of ribosome assembly and protein translation processes that were enriched in the high feed efficiency (FE) pedigree male broiler phenotype**. Components shown in pink or red were enriched in the high FE phenotype (see Table 4). RNA polymerases initiate transcription in the nucleoplasm or in the nucleolus. Small nuclear (sn) ribonucleoproteins (SNP) and heterogeneous (hn) SNP play roles in nucleolar ribosomal RNA (rRNA) synthesis as well as in messenger RNA (mRNA) synthesis in conjunction with spliceosome activity responsible for cleaving on introns from the pre mRNA molecule. Pre-ribosomes as well as tRNAs and mRNAs are exported out of the nucleus into the cytoplasm. In the cytoplasm, ribosomes assemble into the 40S and 60S subunits where protein translation takes place in conjunction with delivery of amino acids by tRNA (See text for details). The figure is adapted from Figure 1 (p. 702) of Wahl et al. (2009).

In this study, we have taken an approach in investigating global gene and protein expression datasets to answer essentially “yes-no” questions; For a given cellular process (e.g., ribosome assembly), are there greater numbers of genes or proteins expressed at higher levels (enriched) in the high or low FE phenotype? This analysis builds upon a series of studies that have been conducted with this line of PedM broilers individually phenotyped for FE (reviewed in Bottje and Carstens, 2009; Bottje and Kong, 2013). These studies pointed toward mitochondria playing an important role in FE in this line of birds on several fronts: (1) higher electron transport chain coupling and less proton leak in high FE mitochondria, and (2) increased reactive oxygen species production in low FE mitochondria. With evidence that the mitoproteome was enriched in the high FE phenotype (Kong B. et al., 2016; Bottje et al., 2017), we investigated mechanisms associated with mitochondrial and cytosolic ribosomal assembly determined that both were enriched in breast muscle of the high FE compared to the low FE PedM broiler phenotype (Figure 1, Tables 2, 3). Since the mitoproteome is comprised mainly of n-DNA encoded proteins, full mitoproteome expression depends on a large contingent of cytosolic translated proteins. The requisite architecture for cytosolic ribosomal assembly and translation was also enriched in the high FE phenotype (Table 4, Figure 2). It should be noted that the expression of many enzymes in nucleic and ribonucleic acid synthesis, including rate limiting steps, were significantly elevated in the high FE phenotype (Bottje and Kong, 2017). Additionally, enrichment of nuclear transport proteins responsible for maintaining separate nuclear and cytosolic compartments and regulating movement of molecules in and out of the nucleus were enriched in the high FE phenotype (Table 4, Figure 2). Enrichment of cytoskeletal/muscle fibers was observed in the low FE phenotype that we have previously hypothesized could require increased energy expenditure in low FE and contribute to the phenotypic expression of low FE (Kong et al., 2011). Differences in enrichment of phosphorylation and kinase families were also observed between the high and low FE phenotypes (Table 5). Finally, quality control processes carried out by proteosomes and autophagy were enriched in the high FE phenotype leading us to speculate that cellular efficiency may involve energy expenditure for degradation and resynthesis in order to maintain optimal protein and organelle function (Table 5). We have also observed up-regulation of mitochondrial adenine nucleotide translocase and voltage dependent activated channel that would facilitate phosphate transfer out of the mitochondria to replenish ATP via the creatine-phosphocreatine shuttle system in the high FE phenotype (Bottje et al., 2017). This creatine-phosphocreatine shuttle could presumably provide a means of continual energy supply needed for resynthesis of proteins and organelles degraded by the proteosome and autophagy systems; the payback to the cell being maintenance of optimal overall cell function.

It is apparent that feed efficiency is a very complex genetic trait. Development of an accurate biomarker, or biomarker panel would be very desirable to aid genetic selection programs. As difficult and elusive as this may be, we hope that this study will add to overall understanding of feed efficiency at the cellular level and will help point researchers in the right direction—whatever that might be. In this report we feel we have provided a convincing argument that there is a functional genomic architecture that would foster transcription and translation processes in the high FE phenotype. Trying to fit a biomarker tool onto this area of cellular metabolism will be difficult due to the inherent complexity. Nevertheless, when effective tools of biomarker selection are developed and implemented, it will help the world in meeting the increasing demands for animal protein that that are with us now, and will only be greater in the future.

## Author contributions

WB, BK, and NH conceived, designed, and conducted the experiments. Data analysis was conducted by WB, BK, NH, and AR. The paper was written through contributions and critical review of the manuscript by all authors (WB, KL, AP, SD, AR, NH, and BK).

## Funding

This work is supported by USDA-NIFA (#2013-01953), Arkansas Biosciences Institute (Little Rock, AR) and the Agricultural Experiment Station (University of Arkansas).

### Conflict of interest statement

The authors declare that the research was conducted in the absence of any commercial or financial relationships that could be construed as a potential conflict of interest. The reviewer DC and handling Editor declared their shared affiliation, and the handling Editor states that the process nevertheless met the standards of a fair and objective review.
